# The core populations and co-occurrence patterns of prokaryotic communities in household biogas digesters

**DOI:** 10.1186/s13068-015-0339-3

**Published:** 2015-09-25

**Authors:** Junpeng Rui, Jiabao Li, Shiheng Zhang, Xuefeng Yan, Yuanpeng Wang, Xiangzhen Li

**Affiliations:** Key Laboratory of Environmental and Applied Microbiology, Chengdu Institute of Biology, Chinese Academy of Sciences, Chengdu, 610041 China; Environmental Microbiology Key Laboratory of Sichuan Province, Chengdu Institute of Biology, Chinese Academy of Sciences, Chengdu, 610041 China; Department of Chemical and Biochemical Engineering, College of Chemistry and Chemical Engineering, Xiamen University, Fujian, 361005 China

**Keywords:** Household biogas digesters, Prokaryotic community, Co-occurrence pattern, Manure digestion, Methanogenesis

## Abstract

**Background:**

Household biogas digesters are widely used to harvest energy in rural areas of developing countries. Understanding core prokaryotic communities, their co-occurrence patterns, and their relationships to environmental factors is important to manage these small-scale anaerobic digestion systems effectively. In this study, 43 household biogas digesters were collected across eight provinces in China. Prokaryotic communities were investigated using 454 pyrosequencing of 16S rRNA genes.

**Results:**

Fourteen core genera and ten core OTUs were identified in household biogas digesters. They were mainly affiliated with the phylum Firmicutes, Synergistetes, Actinobacteria, Chloroflexi, and Spirochaetes. Core prokaryotic genera were mainly composed of *Clostridium*, *Clostridium* XI, *Syntrophomonas*, *Cloacibacillus*, *Sedimentibacter,* and *Turicibacter*. Prokaryotic communities in the 43 samples were clearly divided into two clusters. Cluster I was dominated by *Clostridium*, while Cluster II was dominated by members of Spirochaetes, Bacteroidales, Clostridia, and abundant syntrophs and methanogens. NH_4_^+^-N and COD contributed significantly to the assembly of the prokaryotic community in Cluster I, while NH_4_^+^-N, pH, and phosphate contributed significantly to Cluster II. Correlation-based network analysis showed that the prokaryotic communities in the biogas digesters were dominated by some functional modules. Cluster I was dominated by acetotrophic methanogenic modules and the *Clostridium*-driven primary fermentation module, while the network of Cluster II was dominated by hydrogenotrophic and acetogenic methanogenesis modules and multi-group-driven (Spirochaetes, Bacteroidales, and Clostridia) primary fermentation modules. The network of Cluster II was more complex and functionally redundant.

**Conclusions:**

Prokaryotic communities identified in the household biogas digesters varied significantly and were affected by environmental factors, such as NH_4_^+^-N, pH, and COD. However, core prokaryotic communities existed, and most of them were also dominant populations. Cosmopolitan OTUs tended to co-occur. Prokaryotic communities in biogas digesters were well organized by some functional modules. The modular structure of the prokaryotic community, which has functional redundancy, enhances the resistance against environmental stress and maintains digestion efficiency in the anaerobic digestion process.

**Electronic supplementary material:**

The online version of this article (doi:10.1186/s13068-015-0339-3) contains supplementary material, which is available to authorized users.

## Background

Anaerobic digestion is an effective process for converting organic waste, e.g., animal manure and agricultural or food waste, into biogas containing 50–70 % methane [1, 2]. Generally speaking, digestion consists of four steps: substrate hydrolysis, acidogenesis, acetogenesis, and methanogenesis. The stable and efficient digestion process relies on multiple syntrophic relationships among a community of microbes, including hydrolyzing and fermenting bacteria, acidogenic and acetogenic bacteria, and methanogenic archaea [3, 4]. However, microbial populations in anaerobic manure digesters can be highly variable, even with the digestion of a common core substrate [5]. A deep analysis of the structure and variations of bioreactor microbial communities may potentially reveal their important assembly mechanisms.

Many factors affect the prokaryotic community structure in biogas digesters, including digester design, substrates, and operational conditions [1, 6, 7]. Compared to the large-scale digesters, household biogas digesters are usually small in size that most digesters have volume of less than 10 m^3^. Geographic difference is likely more important to influence anaerobic digestion process in household biogas digesters. For example, temperature is not controlled during the operation; therefore, the digestion process is affected by the seasonal variation of local climate. Mixed raw materials are usually used depending on their local availability, e.g., manures from livestock, humans, and grass residues. Substrate types and quality are often recognized as the primary driving factors shaping microbial communities in anaerobic biogas digesters [8]. As a digester is constantly re-inoculated by multiple substrates, variations in substrate quantity and quality may lead to different microbiomes. Further, microbiomes in the digesters reflect not only the variation of manure quality, but also differences in the digestive tracts of rumen and non-rumen animals. Swine manure is most often used for household biogas digestion in China. It usually contains high ammonium nitrogen (NH_4_^+^-N) due to the high protein content [3]. High NH_4_^+^-N is an inhibitor of methanogenesis, especially acetotrophic methanogens [9]. Therefore, the concentration of NH_4_^+^-N may be a crucial factor affecting prokaryotic community structure in the household biogas digester.

A core OTU is usually defined as being present in most samples [10, 11]. Huse et al. reported that more OTUs will be detected but the differences are minor if using the definition of 90 % prevalence, compared to 95 %. The core microorganisms in this study are defined as those common to most digesters (90 % prevalence), while specific microorganisms exist only in a few or in one digester. The variations in both core and specific populations are related to changes in function (i.e., digestion efficiency) and environmental conditions (i.e., operating conditions). Core microorganisms may have a stronger ability to resist perturbation, while specific microorganisms respond rapidly to some changing conditions. Core and specific microorganisms have been identified, based on seven multiple types of digestion systems, using the clone library method [11]. However, the information is limited by the low throughput clone library method and the small number of digester samples. Moreover, core and specific microbial populations can be better identified by using a high throughput sequencing technique and a larger number of samples from biogas digesters.

The anaerobic methanogenic system is a representative model with a well-organized, closely interacting bacterial and archaeal community. Co-occurrence of prokaryotic populations in the system reflects their similar niche adaptation of the co-occurring species, or interspecies interactions, either by competition or by cooperation. In the anaerobic digestion system, nearly all acidogenic microorganisms also participate in hydrolysis, such as members of *Clostridium*, *Ruminococcus,* and Bacteroidetes [3]. Acetogenesis could be carried out by at least two groups of bacteria: homoacetogens and syntrophs. Acetogenic syntrophs, e.g., the butyrate oxidizer *Syntrophomonas* [12], and the benzoate oxidizer *Syntrophus* [13], can metabolize syntrophically with hydrogenotrophic methanogens. Through the syntrophic metabolism, H_2_ partial pressure is maintained at a very low level to keep anaerobic oxidation of organic matter energetically [4]. Homoacetogens could exergonically produce acetate, competing for substrates with primary fermenters, secondary fermenters, and hydrogenotrophic methanogens [14]. These interactions are also characterized by a co-occurrence network. The correlation-based co-occurrence network analysis can produce microbial functional modules, which enable us to reveal the interactions between different functional groups and environmental factors in various complex systems [15–19].

Household biogas digesters are widely used to harvest energy in rural China and other developing countries [20]. However, according to the literature review, there are few reports using a pyrosequencing technique to compare bacterial communities between various household biogas digesters operated at different geographic locations. The co-occurrence patterns of prokaryotic communities in the household biogas digesters were not revealed. In this study, we collected sludge samples from 43 household biogas digesters across eight provinces of China, and analyzed the variations and co-occurrence networks of prokaryotic communities based on 16S rRNA amplicon pyrosequencing data. The aims were to investigate (1) variations of the prokaryotic community structure, (2) core prokaryotic populations, and (3) the co-occurrence networks of prokaryotic communities in household biogas digesters.

## Results

### Overall prokaryotic community structure and diversity

**Fig. 1:**
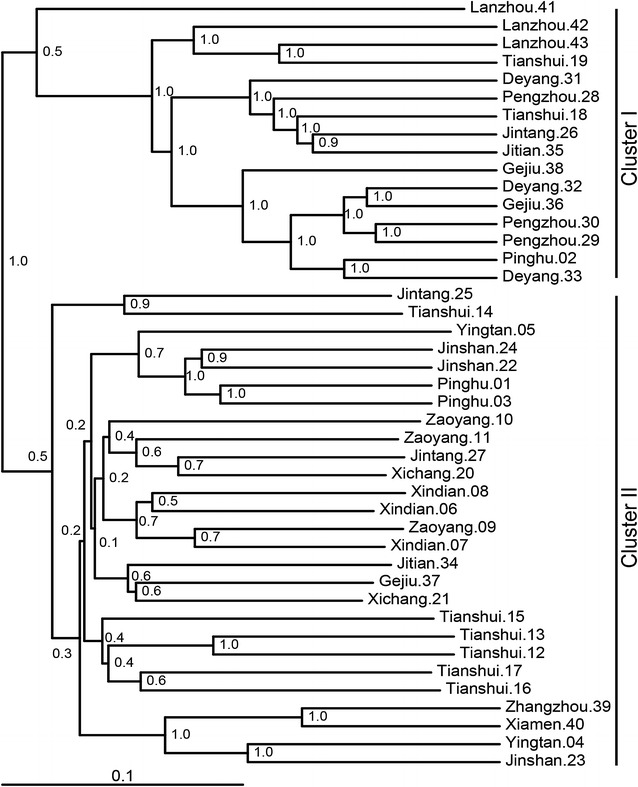
Jackknife sample cluster analysis of prokaryotic communities based on weighted UniFrac distances. The *internal nodes* represent values of Jackknife support

The prokaryotic communities in 43 household biogas sludge samples were separated into two clusters based on UniFrac distances (PerMANOVA *p* < 0.001) (Fig. 1). The prokaryotic communities were clustered independently on substrate types (Additional file 1: Figure S1), but related to different locations. Cluster I contained 16 samples, mainly from Pengzhou, Deyang, Jitian, Gejiu, and Lanzhou. Cluster II contained 27 samples mainly from the remaining 10 rural areas. The prokaryotic diversity indices based on the number of OTUs (operational taxonomic units), Chao1 richness, and Shannon’s and Simpson’s diversity indices, revealed that the prokaryotic diversity of Cluster I was significantly lower than that of Cluster II (*p* < 0.001) (Additional file 1: Figure S2, Additional file 2: Table S1).

**Fig. 2:**
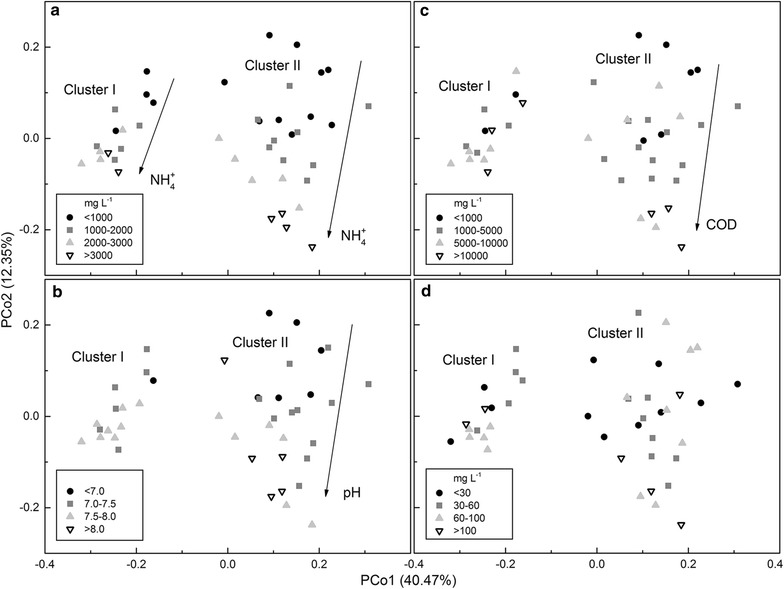
PCoA score plot based on weighted UniFrac metrics. Plots were ranked by the concentrations of **a** NH_4_^+^-N (mg L^−1^), **b** pH, **c** COD (mg L^−1^) and **d** phosphate (mg L^−1^)

The results of principal coordinate analysis (PCoA) showed that the community structures of Cluster I were strongly affected by NH_4_^+^-N, while those of Cluster II were strongly affected by NH_4_^+^-N, COD (chemical oxygen demand), and pH (Fig. 2). Variance partitioning analysis (VPA) was performed to quantify the relative contributions of different environmental variables to changes in the prokaryotic community structure (Additional file 2: Table S2). It showed that COD and NH_4_^+^-N were the primary measured environmental factors to affect community structure in Cluster I, explaining 14.8 and 13.6 % of total observed variation, respectively (*p* < 0.05). NH_4_^+^-N and pH explained 18.9 and 14.4 % of total observed variation in Cluster II, respectively, including 9.0 % shared between them (*p* < 0.01). Therefore, NH_4_^+^-N was the primary environmental factor that influenced community structure in both clusters.

### Core prokaryotic populations in biogas sludge

The 1641 OTUs were detected in these 43 samples based on 97 % identity of 16S rRNA gene sequences. The 961 OTUs were shared between Cluster I and II. A total of 61 OTUs (0.45 % of 1641 OTUs in relative abundance) were detected only in Cluster I, mainly affiliated with Clostridiales. The 619 OTUs (12.31 % of 1641 OTUs in relative abundance) were detected only in Cluster II, mainly affiliated with Bacteroidetes and Spirochaetes. Generally, the OTUs related to *Clostridium*, *Clostridium* XI, *Turicibacter*, Ruminococcaceae, and Anaerolinaceae were more abundant in Cluster I, while those affiliated with Bacteroidales, *Sphaerochaeta*, *Candidatus* Cloacamonas, Porphyromonadaceae, and *Methanosaeta* were more abundant in Cluster II.

OTUs distributed in >90 % of the 43 digesters were defined as core OTUs in this study. Results showed that there were 10 core OTUs, mainly affiliated with Firmicutes, such as *Clostridium*, *Clostridium* XI, *Syntrophomonas,* and *Turicibacter* (Table 1). Members of *Cloacibacillus* and Anaerolinaceae were also included. Generally, most of the core OTUs were also dominant OTUs with a relative abundance of >1 %, and the sum proportion of them was 45.1 and 16.1 % in Cluster I and II, respectively.

**Table 1:** Core genera and core OTUs and their average relative abundances in household biogas digesters

Core genera/OTUs	Relative abundance (%)			Core genera/OTUs	Relative abundance (%)		
	Cluster I	Cluster II	All samples		Cluster I	Cluster II	All samples
Phylum Firmicutes				Phylum Firmicutes			
*Clostridium*	34.37 ± 4.26	10.56 ± 1.29	19.42 ± 2.49	*Anaerovorax*	0.72 ± 0.14	0.57 ± 0.09	0.62 ± 0.08
OTU1	16.39 ± 2.63	5.27 ± 0.90	9.41 ± 1.38	*Ruminococcus*	0.45 ± 0.13	0.66 ± 0.14	0.58 ± 0.10
OTU1200	3.27 ± 0.62	0.59 ± 0.12	1.59 ± 0.31	*Oscillospira*	0.09 ± 0.02	0.32 ± 0.10	0.23 ± 0.06
OTU24	1.29 ± 0.28	0.51 ± 0.14	0.80 ± 0.15	Phylum Synergistetes			
*Clostridium* XI	10.36 ± 1.84	2.09 ± 0.28	5.17 ± 0.92	*Cloacibacillus*	3.90 ± 1.74	4.05 ± 1.13	3.99 ± 0.93
OTU2	5.22 ± 1.17	1.34 ± 0.22	2.78 ± 0.53	OTU5	3.38 ± 1.58	3.31 ± 1.07	3.34 ± 0.87
OTU3	4.62 ± 0.95	0.70 ± 0.12	2.16 ± 0.46	*Aminobacterium*	0.52 ± 0.14	0.85 ± 0.22	0.73 ± 0.15
*Turicibacter*	4.53 ± 0.85	1.57 ± 0.24	2.67 ± 0.41	Phylum Actinobacteria			
OTU4	4.53 ± 0.85	1.57 ± 0.24	2.67 ± 0.41	*Corynebacterium*	0.34 ± 0.16	0.69 ± 0.20	0.56 ± 0.14
*Syntrophomonas*	2.01 ± 0.66	2.19 ± 0.38	2.12 ± 0.33	*Leucobacter*	0.42 ± 0.14	0.09 ± 0.02	0.21 ± 0.06
OTU9	1.63 ± 0.62	1.08 ± 0.31	1.28 ± 0.30	Phylum Spirochaetes			
*Sedimentibacter*	0.44 ± 0.12	1.50 ± 0.35	1.10 ± 0.23	*Candidatus* Cloacamonas	0.39 ± 0.19	5.35 ± 1.02	3.50 ± 0.74
OTU19	0.32 ± 0.08	1.07 ± 0.26	0.79 ± 0.17	Phylum Chloroflexi			
*Tissierella*	1.73 ± 0.48	0.54 ± 0.15	0.98 ± 0.21	OTU10 (Anaerolinaceae)	4.41 ± 1.44	0.67 ± 0.15	2.06 ± 0.59

OTUs distributed in >90 % of samples in each cluster were defined as sub-core OTUs excluding core OTUs. Fourteen sub-core OTUs were identified in Cluster I. They were mainly affiliated with Firmicutes (such as *Clostridium*, *Trichococcus* and Lachnospiraceae), Actinobacteria (*Cloacibacillus*, *Leucobacter*), and the aerobic *Acinetobacter*. These 14 OTUs were also presented in many Cluster II samples, but they were less abundant than those in Cluster I (2.3 vs. 6.4 %) (Additional file 2: Table S3).

Fourteen sub-core OTUs were also identified in Cluster II. They were mainly affiliated with Bacteroidetes (such as Bacteroidales and Porphyromonadaceae) and Spirochaetes (such as *Sphaerochaeta*, *Candidatus* Cloacamonas, and *Treponema*) (Additional file 2: Table S3). The amount of these 14 OTUs was much lower in Cluster I than in Cluster II (1.1 vs. 9.0 % in total), and some of them were not observed in Cluster I.

The definitions of core genera and sub-core genera were similar to those of core OTUs and sub-core OTUs. The 14 core genera were identified, and they were affiliated with Firmicutes, Synergistetes, Actinobacteria, and Spirochaetes. Among them, six core genera contained core OTUs (Table 1). Three sub-core genera in Cluster I and seven in Cluster II (Additional file 2: Table S3) were also identified. The communities of Cluster I mainly consisted of core genera (60.3 % in total), while those of Cluster II mainly consisted of core (31.0 %) and sub-core genera (10.7 %), indicating that prokaryotic communities were more diverse in Cluster II than in Cluster I.

**Fig. 3:**
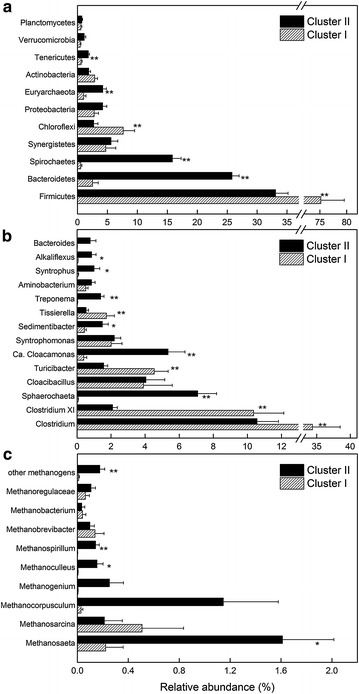
Taxonomic compositions of microbial communities in Cluster I and II. Relative abundances (% of total reads) of 16S rRNA gene **a** at the phylum level of prokaryote, **b** at the genus level of bacteria, and **c** at the genus/family level of methanogens. **Significant at *p* < 0.01, *significant at *p* < 0.05

At the phylum level, Firmicutes were most abundant in both Cluster I and II. Compared to Cluster II, Cluster I digesters had more abundant Firmicutes (75.2 vs. 33.1 %) and Chloroflexi (7.6 vs. 2.7 %), and less abundant Bacteroidetes (2.5 vs. 25.8 %), Spirochaetes (0.5 vs. 15.8 %), Euryarchaeota (1.0 vs. 4.2 %), and Tenericutes (0.6 vs. 1.8 %) (*p* < 0.01, Fig. 3a, Additional file 2: Table S4).

At the genus level, *Clostridium* was most abundant in both Cluster I and II. Compared to Cluster II, Cluster I digesters had more abundant *Clostridium* (34.4 vs. 10.6 %), *Clostridium* XI (10.4 vs 2.1 %), *Turicibacter* (4.5 vs. 1.6 %), and *Tissierella* (1.7 vs. 0.5 %), and less *Sphaerochaeta* (0.07 vs. 7.1 %), *Candidatus* Cloacamonas (0.4 vs. 5.3 %), and *Treponema* (0.04 vs. 1.4 %) (*p* < 0.01, Fig. 3b, Additional file 2: Table S4). The relative abundances of *Cloacibacillus* (about 4 %) and *Syntrophomonas* (about 2 %) were similar in both clusters.

Methanogens were more abundant in Cluster II than in Cluster I (3.9 vs. 1.0 % in total reads, *p* < 0.01). Compared to Cluster I, Cluster II contained more *Methanosaeta* (1.61 vs. 0.22 %, *p* < 0.05), *Methanoculleus* (0.16 vs. 0.008 %, *p* < 0.05), and *Methanospirillum* (0.14 vs. 0.003 %, *p* < 0.01) (Fig. 3c). Besides, *Methanosarcina*, *Methanocorpusculum,* and *Methanogenium* were also abundant in several samples. In general, acetotrophic (*Methanosaeta*, *Methanosarcina*) and hydrogenotrophic methanogens (*Methanocorpusculum*, *Methanogenium*, *Methanoculleus*, *Methanospirillum*, *Methanobrevibacter*, etc.) accounted for 46 and 54 % of all methanogens in both clusters, respectively, without a significant difference.

### Relationships of prokaryotic communities with environmental factors

Pearson’s correlation analysis indicated that the relative abundance of phylum Firmicutes was significantly correlated to phosphate concentration in Cluster I, while it was significantly correlated to pH in Cluster II (Additional file 2: Table S5). Euryarchaeota (e.g. *Methanosaeta*) and *Syntrophus* were negatively correlated with NH_4_^+^-N, indicating that they were sensitive to NH_4_^+^-N (Additional file 1: Figure S3). However, NH_4_^+^-N was positively correlated to Spirochaetes and Tenericutes in Cluster II (*p* < 0.01).

Generally, more genera were significantly correlated to COD and NH_4_^+^-N in Cluster I, while more were significantly correlated to pH, NH_4_^+^-N, and phosphate in Cluster II (Additional file 2: Table S5). *Sphaerochaeta* showed a significant positive correlation with NH_4_^+^-N, COD, phosphate, and pH in Cluster II. In Cluster II, the genus *Clostridium* showed positive correlations, while *Syntrophus* showed negative correlations to pH and NH_4_^+^-N.

The dominant acetotrophic methanogens (genus *Methanosaeta*) and hydrogenotrophic methanogens (especially Methanoregulaceae) were significantly and negatively correlated with both NH_4_^+^-N and pH, while *Methanocorpusculum* was only negatively correlated with pH (*p* < 0.05, Additional file 2: Table S6). In contrast to other methanogens, *Methanoculleus* were positively correlated with both NH_4_^+^-N and pH (*p* > 0.05). COD was negatively correlated with *Methanosaeta* and Methanoregulaceae, while positively correlated with *Methanobrevibacter* (*p* < 0.05). These results indicated that different methanogens were susceptible to different environmental factors.

### Network analysis of cosmopolitan OTUs

Cosmopolitan OTUs were defined as OTUs that occurred in more than half of the samples in the sample group. Cosmopolitan OTUs were identified in Cluster I, II, and in all samples. Nonrandom co-occurrence patterns were detected by the C-score test, with the observed C-scores (6.78, 24.29, and 65.30, respectively) being higher than the mean values (6.65, 23.56, and 63.42 respectively, *p* < 0.0001) expected under the null model, indicating that these cosmopolitans tended to co-occur more often than expected by chance.

**Fig. 4:**
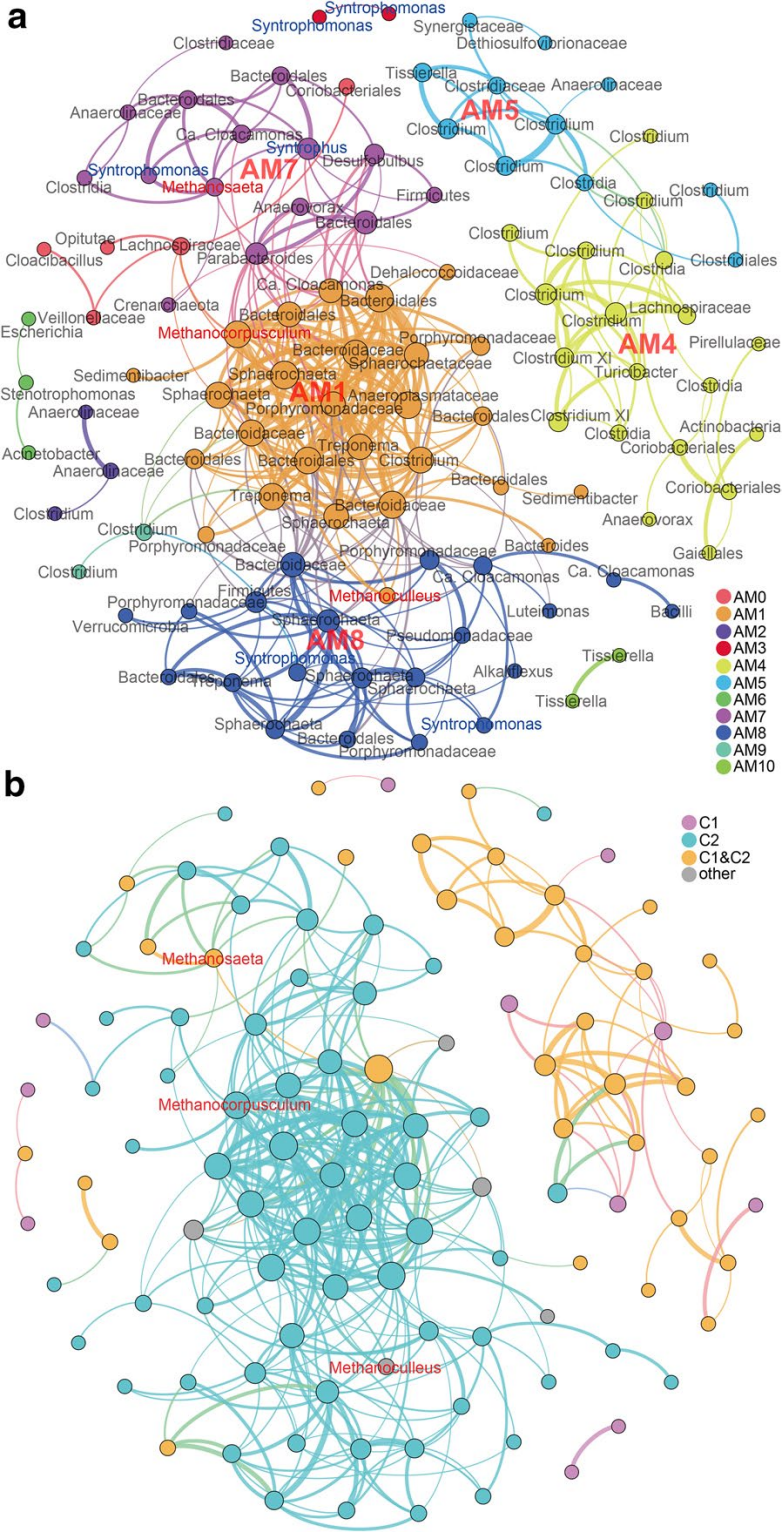
Networks of co-occurring prokaryotic OTUs in all sludge samples based on correlation analysis. Nodes were colored by **a** modularity class with labeled genera names, and **b** occurrence in networks of Cluster I (C1) and Cluster II (C2). A connection stands for a strong (Spearman’s *ρ* > 0.6) and significant (*p* < 0.01) correlation. For each panel, the size of each node is proportional to the number of connections (degree); the thickness of each connection between two nodes (edge) is proportional to the value of Spearman’s correlation coefficients, ranging from 0.60 to 0.93. Other: OTUs did not occur in networks of Cluster I or II

Three correlation-based networks, named C1, C2, and AS, were constructed with these cosmopolitan OTUs for Cluster I, Cluster II, and all samples, respectively (Fig. 4, Additional file 1: Figure S4). Prokaryotic communities in Cluster II digesters showed different topological properties of co-occurring networks from those in Cluster I digesters (Additional file 2: Table S7). The network sizes were similar in AS and C1 (110 and 103 nodes respectively), but were much smaller than C2 (206 nodes). The total abundance of OTUs that occurred in these networks was 60.4, 73.0, and 65.8 %, respectively, indicating that most microorganisms in the sludge samples were affiliated with these cosmopolitan OTUs. Values of modularity, average clustering coefficient, and average path length in these empirical networks were higher than those in random networks, suggesting that the empirical networks had “small world” modularity and hierarchy properties [17, 21].

An important function of each module can be inferred based on the prokaryotic composition, PICRUSt prediction, and their known physiological functions [3, 22]. Cluster I contained eight modules, in which the function of five modules could be predicted confidently. 93 nodes (OTUs) belonged to the module C1M0, 1, 2, 4, and 6 (Table 2, Additional file 2: Table S8), mainly affiliated with Firmicutes (57.9 %), Chloroflexi (6.3 %), and Spirochaetes (4.2 %). The ammonium-sensitive methanogen *Methanosaeta* was in the module C1M1, while *Methanosarcina* was in the high NH_4_^+^-N module C1M2 (positive correlation with NH_4_^+^-N) (Table 3). Large modules C1M1 and C1M2 were predicted to be similar in their function, most likely conducting fermentation mainly with acetotrophic methanogens. C1M4 was also a high-NH_4_^+^-N module, dominated by *Clostridium* for fermentation. The small module C1M6 included aerobic or facultative anaerobic Proteobacteria, e.g., *Sphingomonas*, Methylobacteriaceae, and *Acinetobacter*, which were likely involved in organic substrate degradation and oxygen consumption for the maintenance of anoxic environment. C1M4 had a positive relationship to C1M1, C1M2, and C1M6, reflecting their cooperative nature. The negative relationship between C1M1 and C1M2 reflected a certain competition (Additional file 2: Table S9).

**Table 2:** Taxonomic information of dominant modules in the networks of all samples (AS), Cluster I (C1), and II (C2)

	Number of nodes	Module hubs	Methanogens	Abundant phyla/classes
(1) AS	110			
AM1	27	Bacteroidales, *Sphaerochaeta*, *Treponema*, *Methanocorpusculum*, etc.	*Methanocorpusculum*, *Methanoculleus*	Bacteroidetes, Spirochaetes
AM4	19	*Clostridium*, *Clostridium* XI		Clostridia
AM5	11	*Clostridium*		Clostridia
AM7	15	Bacteroidales, Parabacteroides, *Desulfobulbus*, *Syntrophus*	*Methanosaeta*	Bacteroidetes, Spirochaetes, Euryarchaeota
AM8	21	*Sphaerochaeta*, Bacteroidaceae, Porphyromonadaceae		Bacteroidetes, Spirochaetes, Clostridia
(2) C1	103			
C1M0	13	Coriobacteriales, *Leucobacter* etc.		Clostridia, Actinobacteria, Synergistetes
C1M1	28	*Clostridium*, Pirellulaceae, Gaiellales, etc.	*Methanosaeta*	Clostridia, Chloroflexi, Actinobacteria
C1M2	24	*Clostridium*, Ruminococcaceae, Erysipelotrichaceae	*Methanosarcina*	Clostridia
C1M4	18	*Clostridium*, Clostridia, Coriobacteriales, *Syntrophomonas*		Clostridia
C1M6	10	*Sphingomonas*		Alphaproteobacteria, Gammaproteobacteria
(3) C2	206			
C2M0	9			Clostridia
C2M1	36	*Sphaerochaeta*, Bacteroidales, *Clostridium*, *Tissierella*, *Treponema*, *Cloacibacillus*, etc.		Spirochaetes, Bacteroidetes, Clostridia
C2M2	60	Coriobacteriales	*Methanocorpusculum*	Clostridia, Bacteroidetes, Spirochaetes
C2M4	9			Clostridia, Sphaerochaeta, Bacilli, Actinobacteria
C2M5	29	Bacteroidales, *Syntrophus*, *Syntrophomonas*, *Treponema*	*Methanosaeta*	Spirochaetes, Bacteroidetes, Deltaproteobacteria, Clostridia, Chloroflexi, Euryarchaeota
C2M6	22		*Methanogenium*	Clostridia, Spirochaetes, Bacteroidetes
C2M8	33			Clostridia, Spirochaetes, Bacteroidetes

**Table 3:** Spearman’s correlation of environmental variables to prokaryotic community structures of dominant modules in networks tested by partial Mantel test (permutations: 9999)

	pH	Phosphate	NH_4_^+^-N	COD
(1) All samples	0.223**	0.046	0.264**	0.162*
AM1	0.056	0.111	0.021	0.138*
AM4	−0.011	−0.026	0.012	0.006
AM5	0.244**	−0.026	0.246**	−0.07
AM7	0.147*	0.139*	0.347**	0.323**
AM8	0.059	−0.026	0.110*	0.044
(2) Cluster I	0.165	0.162	0.331*	0.223
C1M0	0.263*	0.069	0.135	0.132
C1M1	0.027	0.033	0.194	0.117
C1M2	0.084	−0.065	0.401**	0.394**
C1M4	0.206*	0.309**	0.300*	0.177
C1M6	−0.084	0.330**	0.067	0.123
(3) Cluster II	0.398**	0.02	0.384**	0.157
C2M0	0.061	−0.051	0.03	−0.083
C2M1	0.295**	−0.058	0.317**	−0.04
C2M2	0.402**	0.194*	0.487**	0.203*
C2M4	0.043	−0.095	−0.048	0.029
C2M5	0.167*	−0.038	0.303**	0.323**
C2M6	0.081	−0.158	0.009	0.1
C2M8	0.164	0.004	0.184*	0.125

** Significant at *p* < 0.01, * significant at *p* < 0.05

When one environmental variable was analyzed by the partial Mantel test, the remaining three environmental variables were controlled

In the 10-module network C2, 198 nodes belonged to module C2M 0, 1, 2, 4, 5, 6, and 8 (Additional file 2: Table S10), which were composed mainly of Firmicutes (22.4 %), Bacteroidetes (19.1 %), and Spirochaetes (13.6 %). Among them, 7 functional modules were identified in C2, including three methanogenic fermentation modules. C2M2 and C2M6 were hydrogenotrophic methanogenic modules, with *Methanocorpusculum* and *Methanogenium* as the key methanogen, respectively. C2M5 was an acetotrophic methanogenic module, with *Methanosaeta* as the key methanogen. C2M1 and C2M8 were primary fermentation modules, including abundant Spirochaetes, Bacteroidetes, and Clostridia. These modules were likely regulated by NH_4_^+^-N. Based on the relationships between its members and NH_4_^+^-N, it is inferred that C2M1 preferred high NH_4_^+^-N, while C2M2, 5, and 8 preferred low NH_4_^+^-N.

C1 and C2 shared 53 nodes distributed in almost all modules. They were mainly affiliated with Clostridia and Anaerolinaceae (Additional file 2: Tables S8, S11). The remaining 53 nodes in C1 were mainly related to Clostridia. However, most of nodes in C1M6 belonged to aerobic Proteobacteria, such as *Methylobacterium* and *Sphingomonas*. The remaining 153 nodes in C2 were mainly affiliated with Spirochaetes and Bacteroidales, and they also included nodes belonging to hydrogenotrophic methanogens (*Methanocorpusculum* and *Methanogenium*) and syntrophs (*Syntrophus* and *Syntrophomonas*).

In the 11-module network AS, 93 nodes belonged to module AM1, 4, 5, 7, and 8 (Additional file 2: Table S11). These nodes were mainly affiliated with Firmicutes (relative abundance of 31.8 % in total OTUs), Bacteroidetes (9.8 %), and Spirochaetes (7.5 %). Hydrogenotrophic methanogens *Methanocorpusculum* and *Methanoculleus* were in AM1, and acetogenic *Methanosaeta* was in AM7. They mainly co-occurred with Bacteroidetes and Spirochaetes (Table 2). Members of AM4 and AM5 were mainly affiliated with Clostridia, especially the genus *Clostridium*. The network AS was a combination of C1 and C2, sharing 105 nodes with them (Additional file 1: Figure S5). Nodes in AM1, 7, and 8 were mainly shared with those in C2, while those in AM4 and 5 were mainly shared with both C1 and C2 (Fig. 4b).

Totally, 388, 330, and 771 pairs of nodes were positively correlated in AS, C1, and C2, respectively (Spearman’s *ρ* > 0.6, *p* < 0.01, Fig. 4), while only 46, 135, and 108 pairs of nodes were negatively correlated, respectively (Spearman’s *ρ* < −0.6, *p* < 0.01, not shown as edges in Fig. 4). Negative associations usually existed between *Clostridium* and Bacteroides/*Syntrophus* in AS, and *Clostridium* and Coriobacteriales/*Facklamia*/*Cloacibacillus*/Anaerolinaceae in C1, probably due to the high abundances of *Clostridium* in many samples (tradeoff or competition interactions). However, negative associations were more complex in C2 since the amount of *Clostridium* was much less in these samples. Negative associations usually existed between *Clostridium*/*Tissierella*/Bacteroidales and *Syntrophus*/*Treponema*, *Sphaerochaeta,* and *Syntrophus*/Bacteroidales in C2.

OTU23 (*Methanocorpusculum*), OTU142 (*Methanoculleus*), OTU14 (*Methanosaeta*) were three nodes belonging to methanogens in AS, with 20, 7, and 7 co-occurrents, respectively (Fig. 4). Their co-occurrents were mainly affiliated to Bacteroidetes and Spirochaetes, which were likely involved in hydrolysis and acidogenesis, and the production of precursors for methanogenesis. OTU14 also co-occurred with acetogenic syntrophs, e.g., *Syntrophomonas* and *Syntrophus*. Therefore, these co-occurrence relationships may reflect a food chain cascade or syntrophic interactions in the anaerobic digestion.

The partial Mantel test showed that NH_4_^+^-N was significantly related to many modules in these three networks, such as AM5, 7 and 8, C1M2 and 4, C2M1, 2, 5, and 6 (Table 3). Modules significantly related to pH or COD were almost related to NH_4_^+^-N as well. It was likely that nodes often shared among modules have the same ammonium preference in different networks. Therefore, NH_4_^+^-N may be an important environmental factor in influencing microbial modularity.

## Discussion

### Core prokaryotic communities in the biogas digesters

In this study, 14 core genera were identified, mainly affiliated with the phylum Firmicutes (9 genera), such as *Clostridium*, *Clostridium* XI, *Syntrophomonas*, *Sedimentibacter,* and *Turicibacter*. The others were affiliated with the phyla Synergistetes (*Cloacibacillus* and *Aminobacterium*), Actinobacteria, and Spirochaetes (*Candidatus* Cloacamonas). Ten core OTUs were identified, mainly affiliated with Firmicutes, such as *Clostridium*, *Clostridium* XI, *Syntrophomonas* and *Turicibacter, Cloacibacillus,* and Anaerolinaceae. Generally, most of the core OTUs were also dominant OTUs in biogas digesters, indicating their importance in biogas fermentation, regardless of the treatment process and geographic locations.

Core genera or OTUs identified in this study are also widely detected in various anaerobic digestion systems [11, 23]. Phylogeny-based empirical relationships can yield powerful correlations between community structure and function as observed in previous studies [24]. Core populations identified in this study have been recognized to play important roles in hydrolysis, fermentation, and syntrophic metabolism. The Genus *Clostridium* participates in both hydrolysis and acidogenesis, and it is especially dominant in the first two digestion phases. The *Clostridium* members decompose various substrates, such as starch, cellulose, amino acids, and fatty acids [3]. Members of *Clostridium* and Bacteroidetes are able to hydrolyze proteins to amino acids with proteases, and degrade amino acids to fatty acids and NH_4_^+^-N [22]. *Clostridium* XI was more abundant in Cluster I. It is affiliated with the family Peptostreptococcaceae, which can ferment saccharides, alcohol, and cellulose [25]. *Sphaerochaeta* was more abundant in Cluster II, which could enhance the degradation of cellulose when grown in co-culture with *Clostridium thermocellum* [26]. *Cloacibacillus* could ferment amino acids (e.g., mucin in swine intestinal tract), and produce fatty acids [27]. *Turicibacter* is able to degrade carbohydrates, which is an important member of the gut microbiota [28]. Anaerolinaceae members were more abundant in Cluster I, and they could ferment carbohydrates and produce hydrogen and acetate [29]. The PICRUSt prediction further supported that the genes encoding enzymes involved in polysaccharides hydrolysis existed in some core populations such as *Clostridium*, *Clostridium* XI, *Sphaerochaeta*, *Leucobacter*, *Turicibacter*, Bacteroidetes, and Anaerolinaceae. Some of them also include genes encoding proteases, such as *Clostridium*, *Clostridium* XI, *Sphaerochaeta*, *Candidatus* Cloacamonas, Bacteroidetes, and Anaerolinaceae.

Each full-scale bioenergy system has a unique community structure with an unprecedented level of stability [24]. Core bacterial populations must be key players in maintaining the stability and function of an anaerobic digestion system. Bacterial community structures are resilient, and key populations will be rebounded following disturbances [24]. The aim of this study is to compare the general assembly rules of microbial community across different digesters. Thus, although we only collected a one-time sample from each of 43 digesters, it may represent the properties of a bacterial community structure in a specific biogas digester.

### Significant variation of prokaryotic community

In this study, the prokaryotic communities of 43 mesophilic household biogas sludge samples were clearly divided into two clusters based on the UniFrac distances, independent of substrate types (Additional file 1: Figure S1B) or our measured environmental factors (pH, COD, NH_4_^+^-N, and phosphate, *p* > 0.05). This indicated the different key factors in shaping the assemblies of prokaryotic communities. Previous work indicated that the prokaryotic communities of 19 full-scale anaerobic digestion installations were divided into two clusters driven by NH_4_^+^-N concentration [30]. The low NH_4_^+^-N cluster was dominant with Bacteroidales, while the high NH_4_^+^-N cluster was dominant with Clostridiales. In this study, we observed more aerobic microbial organisms (e.g., *Sphingomonas* and *Pseudomonas*) and less abundant methanogens in Cluster I digesters. It might be caused by the recent re-inoculation or other disturbance to the digester system. These results possibly implicated poor performance in Cluster I digesters [31]. *Clostridium* was the main primary fermenter in Cluster I digesters, while more diversified primary fermenters occurred in Cluster II digesters, including Spirochaetes, Bacteroidetes, and Clostridia. The abundances of these bacteria were highly correlated with those of methanogens (Table 4).

**Table 4:** Cosmopolitan methanogen OTUs and their significant (*p* < 0.01) co-occurrent OTUs in all samples

Cosmopolitan	Co-occurrents	Spearman’s *ρ*	Co-occurrent affiliations	Co-occurrent affiliated phylum	Number of co-occurring samples
(a) OTU23 (*Methanocorpusculum*)					
	OTU74	0.675	Parabacteroides	Bacteroidetes	20
	OTU78	0.722	Porphyromonadaceae	Bacteroidetes	23
	OTU1240	0.628	Bacteroidales	Bacteroidetes	20
	OTU128	0.721	Sphaerochaetaceae	Spirochaetes	22
	OTU64	0.786	Bacteroidaceae	Bacteroidetes	23
	OTU38	0.708	*Sphaerochaeta*	Spirochaetes	23
	OTU125	0.746	Anaeroplasmataceae	Tenericutes	22
	OTU416	0.840	*Sphaerochaeta*	Spirochaetes	24
	OTU44	0.720	*Candidatus* Cloacamonas	Spirochaetes	23
	OTU169	0.826	*Treponema*	Spirochaetes	25
	OTU137	0.686	Bacteroidales	Bacteroidetes	23
	OTU48	0.749	Bacteroidaceae	Bacteroidetes	22
	OTU92	0.672	Bacteroidales	Bacteroidetes	20
	OTU339	0.659	Bacteroidales	Bacteroidetes	21
	OTU47	0.661	Lachnospiraceae	Firmicutes	21
	OTU91	0.637	*Treponema*	Spirochaetes	23
	OTU303	0.651	*Anaerovorax*	Firmicutes	20
	OTU844	0.722	*Sedimentibacter*	Firmicutes	24
	OTU8	0.604	Bacteroidales	Bacteroidetes	26
	OTU72	0.859	*Sphaerochaeta*	Spirochaetes	27
(b) OTU14 (*Methanosaeta*)					
	OTU52	0.666	Porphyromonadaceae	Bacteroidetes	25
	OTU96	0.653	*Syntrophus*	Proteobacteria	17
	OTU749	0.657	Bacteroidales	Bacteroidetes	23
	OTU12	0.689	*Candidatus* Cloacamonas	Spirochaetes	17
	OTU87	0.742	*Syntrophomonas*	Firmicutes	28
	OTU66	0.724	Bacteroidales	Bacteroidetes	23
	OTU119	0.703	Clostridia	Firmicutes	23
(c) OTU142 (*Methanoculleus*)					
	OTU327	0.610	Crenarchaeota	Crenarchaeota	22
	OTU8	0.635	Bacteroidales	Bacteroidetes	32
	OTU38	0.619	*Sphaerochaeta*	Spirochaetes	21
	OTU137	0.714	Bacteroidales	Bacteroidetes	23
	OTU169	0.648	*Treponema*	Spirochaetes	22
	OTU91	0.660	*Treponema*	Spirochaetes	22
	OTU454	0.628	*Candidatus* Cloacamonas	Spirochaetes	24

The genus *Syntrophus* is able to syntrophically oxidize benzoate with hydrogenotrophic methanogens, and produce acetate and H_2_ [13]. The genus *Candidatus* Cloacamonas is probably a hydrogen-producing syntroph present in many anaerobic digesters [32]. Both of these genera were significantly higher in Cluster II than in Cluster I, indicating active secondary fermentation in Cluster II digesters. Methanogenic activity appears in the acidogenic phase, but the number of methanogenic archaea obviously increases in the methanogenic phase [3]. Methanogens, especially *Methanosaeta*, *Methanoculleus,* and *Methanospirillum*, were more abundant in Cluster II than in Cluster I (*p* < 0.05), indicating methanogenesis was possibly more active in Cluster II digesters.

### Selective inhibition of NH_4_^+^-N affects prokaryotic community structure

Many environmental factors influence prokaryotic communities in the biogas digestion system, such as substrates, pH, inoculation, etc. [3, 33]. If one environmental factor predominates the microbial community structure, it may decouple the relationships between community structures and other factors. In this study, it is difficult to collect particular data for household biogas digesters, such as gas production rate, hydraulic retention time, exact substrate compositions, and so forth. Among our measured environmental parameters, the NH_4_^+^-N, pH, and COD were observed to strongly influence prokaryotic communities in the household digesters. Phosphate, which was positively correlated to NH_4_^+^-N (*p* < 0.01), had less effect on prokaryotic communities, except for module C1M4 and C1M6 dominant by *Clostridium* and aerobic Proteobacteria, respectively.

Swine manure as a main substrate used in the Chinese household digesters often contains high NH_4_^+^-N. VPA analysis indicated that NH_4_^+^-N is an important factor in influencing the prokaryotic community structure in both Cluster I and Cluster II. High NH_4_^+^-N has an inhibiting effect, and may even be toxic to microbial communities because free ammonia could diffuse passively into cells, causing a proton imbalance and potassium deficiency [34, 35]. High NH_4_^+^ ion (>1500 mg L^−1^ NH_4_^+^-N) also has an inhibiting effect on those species (e.g., methanogens) sensitive to pH [3, 34]. The NH_4_^+^-N concentration of 25 samples were higher than 1500 mg L^−1^ in this study. Compared to bacteria, methanogenic archaea are more susceptible to NH_4_^+^-N. Moreover, the tolerance of hydrogenotrophic methanogens to ammonium is usually higher than that of acetoclastic *Methanosarcina* and *Methanosaeta* [9]. In this study, the relative abundance of Euryarchaeota was negatively correlated with NH_4_^+^-N in both clusters (*p* < 0.05), while only the most dominant methanogen *Methanosaeta* was inhibited in Cluster II (*p* < 0.05). This indicated that the keystone populations can be altered by NH_4_^+^-N. The microbial community may select syntrophic acetate oxidation as a significant pathway for forming methane from acetate under high NH_4_^+^-N concentration [36]. Besides NH_4_^+^-N concentration, the degree of ammonia inhibition could also be influenced by temperature, pH, volatile fatty acids, and some other ions [34]. It is reported that some ions (e.g., Na^+^, Mg^2+^, and Ca^2+^) could be antagonistic to ammonia inhibition [37]. The adaptations of methanogens to ammonia were also observed [38]. The adaptations might be common for the microbial populations due to diverse substrates and long hydraulic retention time in household biogas digesters.

Core methanogen OTU was not observed, indicating that they are susceptible to environmental changes, e.g., NH_4_^+^-N. Besides methanogens, this study observed that some bacteria were also inhibited by NH_4_^+^-N, including Proteobacteria (e.g., *Syntrophus*) and Planctomycetes in Cluster II. However, some bacteria were positively correlated to NH_4_^+^-N, including *Clostridium* and *Sphaerochaeta*, *Erysipelothrix,* and *Tissierella*. Therefore, the selection of different prokaryotic taxa by NH_4_^+^-N would shift the community structure through the adjustment of species abundance (species sorting), in which those species genetically better adapted to high NH_4_^+^-N may outcompete other less well-adapted species.

### Co-occurrence patterns of prokaryotic communities

Co-occurrence network analysis is useful in revealing common system-level properties of prokaryotic communities in the biogas digestion systems. Co-occurrence analysis of microbial taxa from 43 household digesters in this study suggested strong within- and between-domain correlations between different groups of microorganisms within the digesters. It also showed that the prokaryotic communities in biogas digesters are well organized by some functional modules. Significant and positive correlations between members within the modules indicated they may co-occur with mutualism interactions, such as an exchange of metabolic intermediates. Methanogenesis is a central metabolic process in the anaerobic biogas digestion. As abundant methanogens in the household biogas digesters, OTUs affiliated to hydrogenotrophic *Methanocorpusculum* and *Methanoculleus* and acetoclastic *Methanosaeta* tended to co-occur with fermentation bacterial Bacteroidetes, Spirochaetes, Tenericutes, and Firmicutes (Table 1). These bacteria participate in hydrolysis and produce intermediates, e.g., H_2_/CO_2_, formate, and acetate [3]. The occurrence of a modularity structure in the prokaryotic community further indicates the occurrence of multiple syntrophic metabolic pathways with functional redundancy of competition or cooperation populations in the biogas digesters. Besides the exchange of metabolic intermediates, multiple syntrophic interactions must be maintained between bacteria and methanogens, which consume H_2_ and maintain a low H_2_ partial pressure, so that the overall reaction in the system is exergonic [4]. This is further supported by the fact that the positive interactions of multi-group-driven primary fermentation modules with hydrogenotrophic methanogenic fermentation modules were much stronger than those with acetotrophic methanogenic fermentation modules (Additional file 1: Figure S6). The different co-occurrence networks were observed between Cluster I and Cluster II digesters in this study. It was speculated that different co-occurrence networks may influence the stability and performance of biogas digesters.

The assembly of microbial communities is controlled by neutral and deterministic processes [39]. Recent studies indicated that deterministic processes may play a larger role in the process of microbial community assembly in anaerobic digesters [40]. Interspecies interactions and environmental selections are proposed to be two relevant mechanisms of deterministic factors [41, 42]. The integrative effects of these environmental factors may create niche differentiation, and cause the variations in microbial community structure in various digesters. Further, the results in this study showed that cosmopolitan OTUs tended to co-occur, and microbial communities showed modularity properties in the biogas digesters. These modules and their inferred central functions are highly correlated to some environmental factors, e.g., NH_4_^+^-N, pH, and COD. Thus, the modular structure of microbial interactions may be largely shaped by the deterministic processes.

## Conclusions

The present study showed that 14 genera and 10 OTUs of prokaryotic populations were commonly shared by at least 90 % of all 43 samples. They were mainly affiliated with the phyla Firmicutes, Synergistetes, Actinobacteria, Chloroflexi, and Spirochaetes. Core prokaryotic genera were mainly composed of *Clostridium*, *Clostridium* XI, *Syntrophomonas*, *Cloacibacillus*, Anaerolinaceae, *Sedimentibacter,* and *Turicibacter*. Prokaryotic communities of the 43 samples showed high variations and were clearly separated into 2 clusters with different co-occurrence networks. Cluster I was dominated by *Clostridium*, while Cluster II was dominated by members of Spirochaetes, Bacteroidales, Clostridia, and abundant syntrophs and methanogens. NH_4_^+^-N and COD contributed significantly to the assembly of the prokaryotic community in Cluster I, while NH_4_^+^-N, pH, and phosphate contributed significantly to the community assembly in Cluster II. Correlation-based network analysis showed that the prokaryotic communities of biogas digesters are well organized by some functional modules. These modules and their inferred central functions are highly correlated to some environmental factors, such as NH_4_^+^-N, pH, and COD. Anaerobic digestion is susceptible to various forms of perturbation because of its delicate balance between the different microbial consortia in the anaerobic digestion process. The modular structure of the prokaryotic community with functional redundancy in the biogas digestion system may provide the system with access to the total functional diversity and environmental specificity available in the community, thus, enhances the resistance against perturbation, and maintains the performance of biogas digesters.

## Methods

### Sample description and chemical property measurements

Forty-three sludge samples from household biogas digesters were collected in 15 rural areas across eight provinces in China (Additional file 2: Table S12). These digesters, which are also called hydraulic biogas digesters, were typically constructed using brick and concrete in a fixed-dome configuration. All digesters were operated in a temperature range from 18 to 35 °C without temperature control. The volume of most digesters ranged from 6 to 25 m^3^. Only one digester had a volume of 55 m^3^. The feeding substrates varied among individual digesters, including manures from swine, cattle, humans, poultry, and donkeys. Grass residue was used occasionally in some digesters. Usually three bottles of sludge samples from each digester were collected into sterile flasks, transported to the lab under ice, pooled and centrifuged under 8000 rpm, and stored at -20 °C until the genomic DNA were extracted. Chemical properties of sludge, including pH, chemical oxygen demand, NH_4_^+^-N, and phosphate were measured as previously described [43, 44].

### DNA extraction and pyrosequencing

Genomic DNA was extracted by the method described previously [45]. DNA quality was checked using a NanoDrop Spectrophotometer, subjected to electrophoresis, and visualized in a 0.8 % agarose gel. Extracted DNA was diluted to 10 ng μl^−1^ for downstream use. For pyrosequencing, the 16S rRNA gene was amplified with universal primers 515F (5′-GTGYCAGCMGCCGCGGTA-3′) and 909R (5′-CCCCGYCAATTCMTTTRAGT-3′). The detailed PCR conditions were described previously [44]. The barcoded amplicons were pooled with equal molar concentrations of the samples and sequenced using a GS FLX + pyrosequencing system (454 Life Sciences).

### Sequencing data analysis

The raw sequences were sorted based on unique barcodes, trimmed for sequence quality, and clustered at 97 % identity for OTUs with USEARCH v7.0 (http://www.drive5.com/usearch/download.html) using UPARSE pipeline [46]. Chimeras and singletons were removed from clustered sequences with USEARCH. Re-sampling to the same sequence depth (2230 sequences per sample) was performed using daisychopper.pl (http://www.festinalente.me/bioinf/downloads/daisychopper.pl) prior to downstream analysis. Chao1 estimator of richness and Shannon’s and Simpson’s diversity indices were calculated using QIIME pipeline v1.7.0 (http://qiime.org/tutorials/tutorial.html) [47]. The phylogenetic affiliation of each sequence was analyzed by an RDP Classifier at a confidence level of 80 % [48]. Gene functions of dominant OTUs were predicted using PICRUSt [49], a tool that predicts the gene function of a microbial community using an existing database of microbial genomes. It is usually used well in predicting the function of microbiome from simple habitats, such as human and animal gut. Recently it is also used to study soil microbiome [50]. To predict the gene function of an OTU, the OTU representative sequence is assigned to a reference sequence in the GreenGenes database at 97 % identity using QIIME. Then, the functional profile of the reference sequence is found in COG and/or KEGG orthology databases using PICRUSt.

The original pyrosequencing data from this study were available at the European Nucleotide Archive by accession no. PRJEB10542 (http://www.ebi.ac.uk/ena/data/view/PRJEB10542).

### Statistical analysis

Overall structural changes of prokaryotic communities were evaluated by PCoA in Fast UniFrac [51]. The statistical significance among datasets was assessed by PerMANOVA using the weighted PCoA scores in PAST (http://folk.uio.no/ohammer/past/). The partial Mantel test was applied to evaluate the correlations among prokaryotic communities with environmental variables. Variance partitioning analysis (VPA) was performed to quantify the relative contributions of environmental variables based on redundancy analysis (RDA) using the R package Vegan (http://cran.r-project.org/web/packages/vegan/index.html). One-way-analysis of variance (ANOVA), regression and correlation analysis between prokaryotic abundances and environmental factors were conducted using SPSS 21 software.

### Co-occurrence network analysis

OTUs occurred in more than half of samples were used for network analysis. Non-random co-occurrence patterns of selected OTUs were tested with the checkerboard score (*C*-score) under a null model [15, 52]. Spearman’s rank correlations between selected OTUs were calculated [16]. A valid co-occurrence event was considered to be a robust correlation if the Spearman’s correlation coefficient was *ρ* > 0.6 with a significance of *p* < 0.01 [15]. Correlation networks were constructed with the robust correlations as weighted edges using Gephi software (https://gephi.github.io/). 10,000 Erdös-Réyni random networks with the same number of nodes and edges as the empirical networks were generated using the R package igraph (http://cran.r-project.org/web/packages/igraph/) [16].

## Additional files

**Additional file 1: Figure S1.** PCoA score plot based on weighted UniFrac metrics colored by (A) locations, and (B) substrates. P: swine manure; B: cattle manure; H: human manure; C: poultry manure; E: donkey manure; G: grass. **Figure S2.** Rarefaction curve of observed OTUs before re-sampling. **Figure S3.** Relationships between (A) NH_4_^+^-N concentration and the relative abundance of Euryarchaeota in Cluster II, (B) the relative abundance of Clostridium and that of Euryarchaeota, and (C) the relative abundance of Bacteroidetes and that of Spirochaetes in all samples. **Figure S4.** Networks of co-occurring prokaryotic OTUs in (A) Cluster I and (B) Cluster II based on correlation analysis. OTUs were colored by modularity class with labeled genera names. A connection stands for a strong (Spearman’s ρ > 0.6) and significant (*p* < 0.01) correlation. For each panel, the size of each node is proportional to the number of connections (degree); the thickness of each connection between two nodes (edge) is proportional to the value of Spearman’s correlation coefficients ranging from 0.60 to 0.95. Ca.: Candidatus. **Figure S5.** Number of shared nodes (OTUs) among networks AS, C1, and C2. **Figure S6.** Relationships among functional modules of prokaryotic communities of (A) Cluster I and (B) Cluster II. The shapes of each module represent the main function of the module. The color of each module represents the correlation between the module and NH_4_^+^-N concentration: black, positive correlation (*p* < 0.05); white, negative correlation (*p* < 0.05); grey, no significant correlation. The thickness of each solid line between modules is proportional to the sum of positive Spearman’s ρ between them in the networks (C1 and C2) ranging from 0.6 to 18.5; a dotted line represents over 10 couples of OTUs with significant negative correlations (Spearman’s ρ < −0.6, *p* < 0.01) between modules.
**Additional file 2: Table S1.** Prokaryotic diversity indices based on 97 % identity of 16S rRNA gene sequences and 2230 reads per sample. **Table S2.** The relative contributions (R square value) of each environmental factor to OTUs in all samples, Cluster I and II based on RDA analysis. **Table S3.** Relative abundances of core genera/OTUs in all 43 samples and sub-core genera/OTUs in Cluster I and II, and their correlation to environmental factors. **Table S4.** Relative abundances of the abundant phyla (average relative abundance >0.1%) and genera (average relative abundance >0.05 %). **Table S5.** Pearson’s correlation of abundant phyla and genera to environmental factors in Cluster I and II. **Table S6.** Pearson’s correlation of abundant methanogens to environmental factors in all samples. **Table S7.** Topological properties of co-occurring networks AS (43 samples), C1 (27 samples in Cluster I), and C2 (16 samples in Cluster II), generated with Gephi software. **Table S8.** Node information of 103 cosmopolitan OTUs in the network C1 (Cluster I). **Table S9.** Positive and negative interactions among modules in network AS, C1, and C2. **Table S10.** Node information of 206 cosmopolitan OTUs in the network C2 (Cluster II). **Table S11.** Node information of 110 cosmopolitan OTUs in the network AS (all samples). **Table S12.** Fermentation conditions and chemical properties in biogas digesters.

## Abbreviations

ANOVA: analysis of variance
COD: chemical oxygen demand
OTU: operational taxonomic unit
PCoA: principal coordinates analysis
PerMANOVA: permutational multivariate analysis of variance
RDA: redundancy analysis
VPA: variance partitioning analysis
