# Supplementary material for: The core populations and co-occurrence patterns of prokaryotic communities in household biogas digesters
Source: Biotechnol Biofuels. 2015 Sep 25;8:158. doi: 10.1186/s13068-015-0339-3 (PMC4582640; doi:10.1186/s13068-015-0339-3)
Supplement: Supplementary file 1 — Additional file 1: Figure S1. PCoA score plot based on weighted UniFrac metrics colored by (A) locations, and (B) substrates. P: swine manure; B: cattle manure; H: human manure; C: poultry manure; E: donkey manure; G: grass. Figure S2. Rarefaction curve of observed OTUs before re-sampling. Figure S3. Relationships between (A) NH4 +-N concentration and the relative abundance of Euryarchaeota in Cluster II, (B) the relative abundance of Clostridium and that of Euryarchaeota, and (C) the relative abundance of Bacteroidetes and that of Spirochaetes in all samples. Figure S4. Networks of co-occurring prokaryotic OTUs in (A) Cluster I and (B) Cluster II based on correlation analysis. OTUs were colored by modularity class with labeled genera names. A connection stands for a strong (Spearman’s ρ > 0.6) and significant (p < 0.01) correlation. For each panel, the size of each node is proportional to the number of connections (degree); the thickness of each connection between two nodes (edge) is proportional to the value of Spearman’s correlation coefficients ranging from 0.60 to 0.95. Ca.: Candidatus. Figure S5. Number of shared nodes (OTUs) among networks AS, C1, and C2. Figure S6. Relationships among functional modules of prokaryotic communities of (A) Cluster I and (B) Cluster II. The shapes of each module represent the main function of the module. The color of each module represents the correlation between the module and NH4 +-N concentration: black, positive correlation (p < 0.05); white, negative correlation (p < 0.05); grey, no significant correlation. The thickness of each solid line between modules is proportional to the sum of positive Spearman’s ρ between them in the networks (C1 and C2) ranging from 0.6 to 18.5; a dotted line represents over 10 couples of OTUs with significant negative correlations (Spearman’s ρ < −0.6, p < 0.01) between modules. [file 13068_2015_339_MOESM1_ESM.docx]

**Additional file 1**


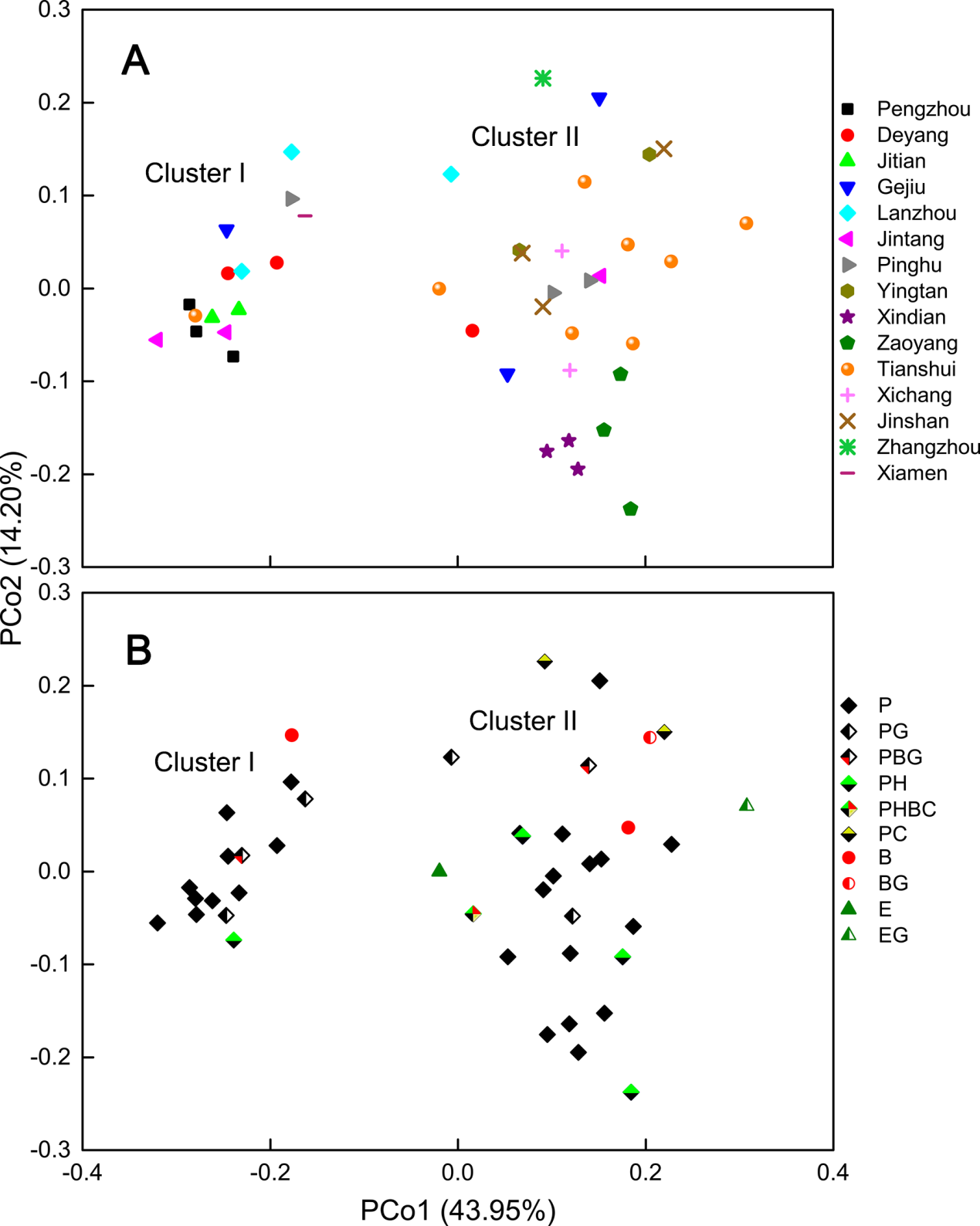


**Figure S1.** PCoA score plot based on weighted UniFrac metrics colored by (A) locations, and (B) substrates. P: swine manure; B: cattle manure; H: human manure; C: poultry manure; E: donkey manure; G: grass.


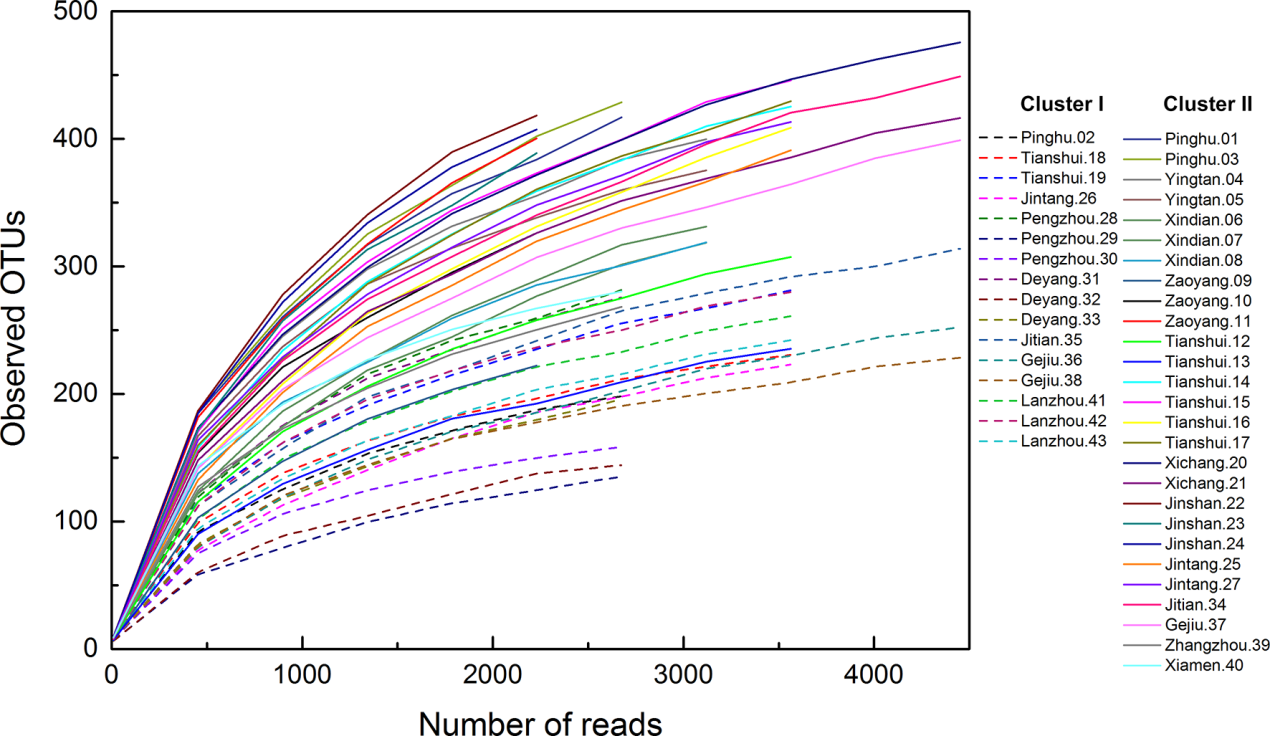


**Figure S2.** Rarefaction curves of observed OTUs before re-sampling.


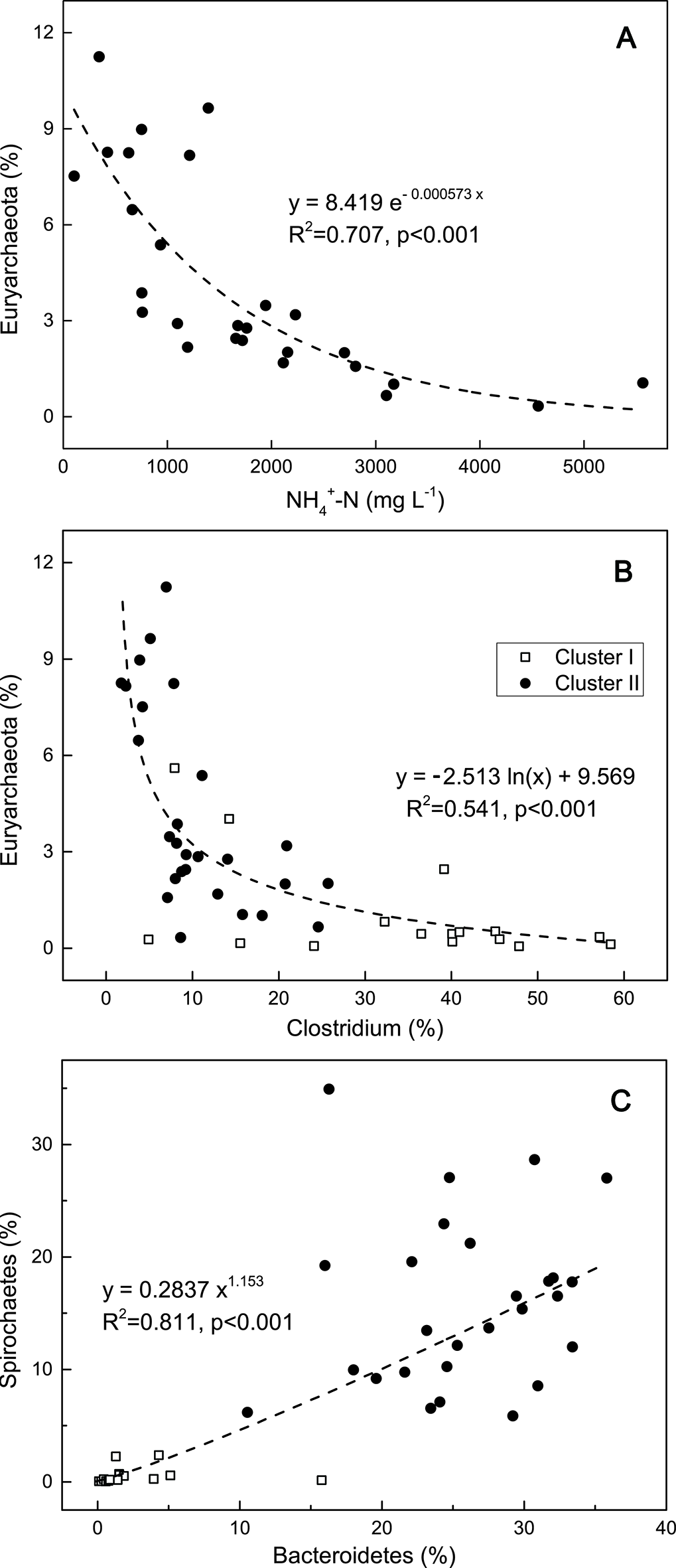


**Figure S3.** Relationships between (A) NH_4_^+^-N concentration and the relative abundance of Euryarchaeota in Cluster II, (B) the relative abundance of Clostridium and that of Euryarchaeota and (C) the relative abundance of Bacteroidetes and that of Spirochaetes in all samples.


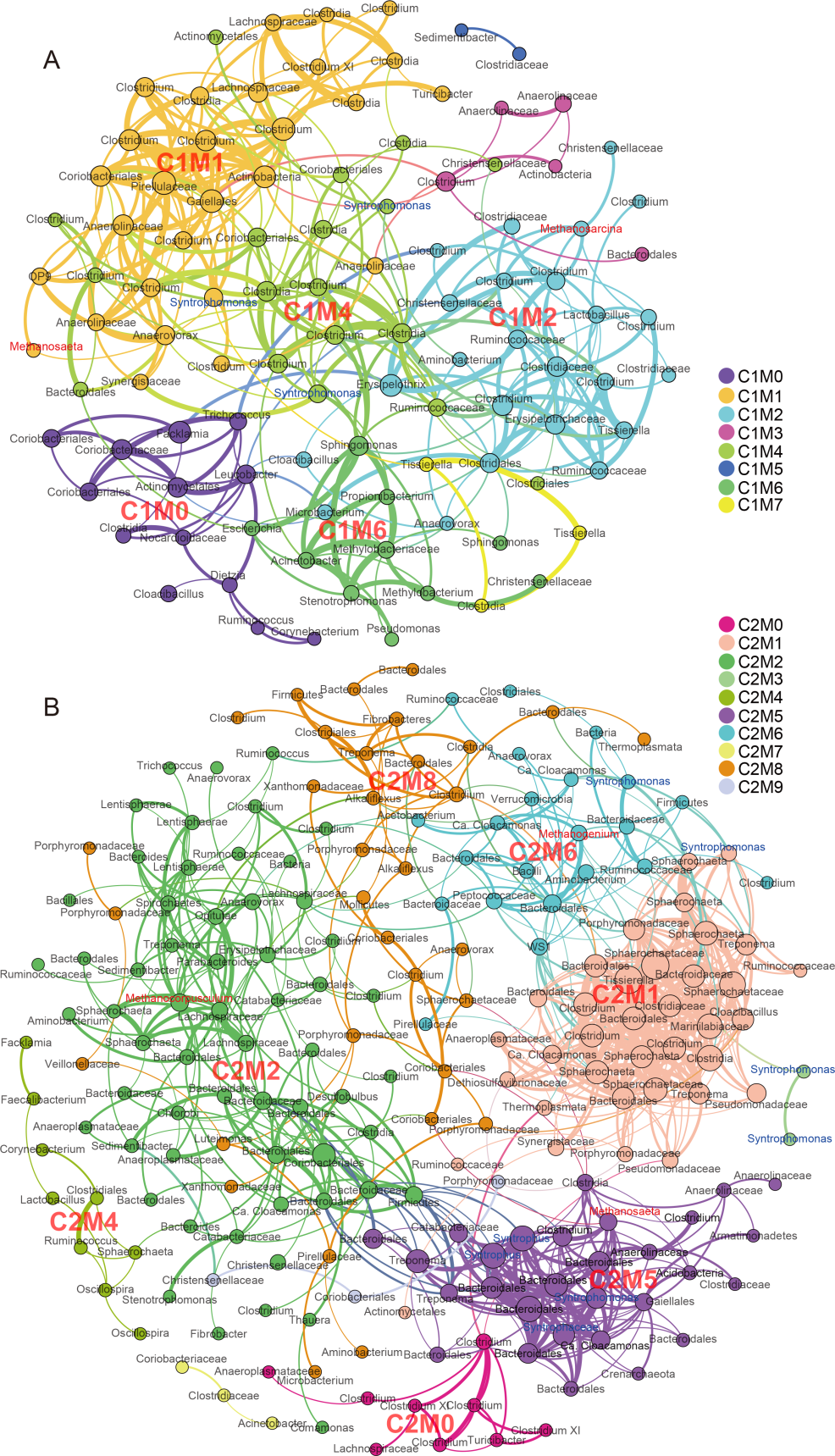


**Figure S4.** Networks of co-occurring prokaryotic OTUs in (A) Cluster I and (B) Cluster II based on correlation analysis. OTUs were colored by modularity class with labeled genera names. A connection stands for a strong (Spearman’s ρ>0.6) and significant correlation (p<0.01). For each panel, the size of each node is proportional to the number of connections (degree); the thickness of each connection between two nodes (edge) is proportional to the value of Spearman’s correlation coefficients ranging from 0.60 to 0.95. Ca.: Candidatus.





**Figure S5.** Number of shared nodes (OTUs) among networks AS, C1 and C2.





**Figure S6**. Relationships among functional modules of prokaryotic communities of (A) Cluster I and (B) Cluster II. The shapes of each module are representative of main function of the module. The color of each module is representative of the correlation between the module and NH_4_^+^-N concentration: black, positive correlation (p<0.05); white, negative correlation (p<0.05); grey, no significant correlation. The thickness of each solid line between modules is proportional to the sum of positive Spearman’s ρ between them in the networks (C1 and C2) ranging from 0.6 to 18.5; a dotted line represents over 10 couples of OTUs with significant negative correlations (Spearman’s ρ<-0.6, p<0.01) between modules.
